# Rehabilitation Exercises Supported by Monitor-Augmented Reality for Patients with High-Grade Glioma Undergoing Radiotherapy: Results of a Randomized Clinical Trial

**DOI:** 10.3390/jcm12216838

**Published:** 2023-10-29

**Authors:** Anna Pieczyńska, Ewa Zasadzka, Agnieszka Pilarska, Danuta Procyk, Krystyna Adamska, Katarzyna Hojan

**Affiliations:** 1 Department of Occupational Therapy, Poznan University of Medical Sciences, 61-781 Poznan, Poland; apieczynska@ump.edu.pl (A.P.); katarzyna.hojan@wco.pl (K.H.); 2 Department of Rehabilitation, Greater Poland Cancer Centre, 61-866 Poznan, Poland; agnieszka.pilarska@wco.pl; 3 Central Laboratory, Greater Poland Cancer Centre, 15, 61-866 Poznan, Poland; danuta.procyk@wco.pl; 4 Chair and Department of Electroradiology, Poznan University of Medical Science, 61-781 Poznan, Poland; krystynaadamska@ump.edu.pl; 5 3rd Radiotherapy Department, Greater Poland Cancer Centre, 61-866 Poznan, Poland

**Keywords:** glioma, exercise, rehabilitation interventions, radiotherapy

## Abstract

Background: Exercise has been shown to improve quality of life (QoL) and even treatment outcomes in cancer patients. However, the evidence to support the benefits of exercise in patients with high-grade glioma (HGG) is limited. Therefore, we performed a randomized clinical trial (RCT) to examine the effect of augmented-reality-based rehabilitation exercises on physical and functional fitness, cognitive function, fatigue, mood, QoL, selected blood parameters, brain derived neurotrophic factor (BDNF), and S100 protein in patients with HGG. Methods: Adult patients with HGG scheduled to undergo radiotherapy after tumor resection were randomized to participate in an exercise program (experimental group, *n* = 25) or to receive usual care (controls, *n* = 22). Physical and mental fitness was measured at baseline, after the completion of radiotherapy, and at 3 months. The following tests were administered: Handgrip Strength Test; 6-Minute Walk Test; Time Up and Go test; Functional Independent Measure scale; Addenbrooke’s Cognitive Examination III (ACE III); Hospital Anxiety and Depression Scale; Functional Cancer Therapy Assessment—Brain; and Functional Assessment of Chronic Illness Therapy—Fatigue. We also measured blood parameters, BDNF, and S100 protein levels. Results: No significant changes were observed in the exercise group. However, the controls experienced a significant decrease in HGS and in the ACE III attention domain. No significant changes were observed in QoL, fatigue, BDNF, or S100 levels in either group. Conclusions: Augmented-reality-based exercise during radiation therapy may prevent loss of muscle strength and attention in patients with HGG.

## 1. Introduction

High-grade glioma (HGG) is an aggressive type of brain tumor. Although effective treatment options remain limited, standard care includes surgical resection, chemotherapy, radiotherapy (RT), and immunotherapy [1]. However, most patients develop recurrent disease. Although complete recovery is generally not possible, treatment can prolong the progression-free survival time [2], but with important treatment-related side effects, which may include motor dysfunction, neurocognitive disorders, pain, and fatigue [3,4], Fatigue is one of the most widespread and distressing long-term effects of cancer, particularly in brain tumor patients (in whom rates are 40–50% higher than in patients with other types of cancer) [5]. The effects of the disease and/or the treatment can result in mobility limitations, the impairment of activities of daily living (ADL), depression, loss of functional independence, and a decrease in quality of life (QoL) [3,6]. For this reason, maintaining or even improving QoL has increasingly become one of the main treatment objectives for these patients [7]. Although QoL in patients with glioblastoma is often already impaired at diagnosis [8], the side effects of treatment may further worsen QoL [9].

In recent years, several literature reviews have shown that physical activity and rehabilitation (both physical and cognitive) can improve motor function, cognitive function, the performance of ADLs, and QoL in patients with HGG [10,11], which is why the European Society of Neuro-Oncology recommends rehabilitation for patients with brain tumors [12]. Research has shown that, during oncological treatment, many patients reduce their physical activity levels [11]. Moreover, patients with brain tumors show lower levels of physical activity (only 22–41% meet recommended levels) than patients with other types of cancer (approximately 50%) [11,13]. Given these data, it could be advantageous to initiate exercise at the hospital during oncological treatment and to continue it after treatment has been completed.

At present, there is little information on the effectiveness of rehabilitation programs specifically designed for people with glioblastoma. However, some studies have been performed to assess the feasibility and effectiveness of resistance and aerobic training in different settings, including outpatient, hospital, home, and virtual reality environments [10,14,15]. To the best of our knowledge, only two studies [16,17] using virtual reality in the treatment of patients with brain tumors have been published so far. However, the results of a meta-analysis [18] indicate the usefulness of physiotherapy using augmented reality in improving balance and gait, upper limb functionality, muscle mass, physical performance, and exercise self-efficacy and in reducing the risk of falls in other patient groups. Augmented reality is increasingly used to support patients in physical rehabilitation, enabling them to perform virtual exercises and track progress in real time [19]. Virtual and augmented reality technology has great potential in the field of neurorehabilitation, providing patients with an engaging and safe environment in which to improve their motor and cognitive functions. This technology offers a more engaging and personalized approach to rehabilitation [19]. In turn, the most successful interventions in the rehabilitation of brain tumor patients reported to date are those that include personalized exercise recommendations, individualized training, and strategies designed to increase adherence, such as close monitoring of training data and regular guidance from a physiotherapist [10]. Given these findings, it seems that exercise and training programs supported by augmented reality could be highly beneficial, as they would allow physiotherapists to personalize the exercise program and to monitor the patient’s performance in the hospital ward and at home [20,21].

Another key aspect of rehabilitation in this patient population (apart from physical exercise) is cognitive function therapy. At diagnosis, a substantial proportion of patients report cognitive impairment (31–81%), at a rate that is higher than in any other type of cancer [22]. The incidence of cognitive dysfunction may vary over the course of the disease. Although some reports suggest that cognitive function improves 3 to 6 months after tumor resection [22], other studies have found that cognitive function remains impaired (or even worsens further) due to radiotherapy and chemotherapy and/or tumor progression. Not surprisingly, this dysfunction also negatively impacts health-related quality of life (HRQoL) for these patients [22,23].

The effectiveness of rehabilitation in patients with primary brain tumors can be assessed through the use of functional assessment scales and fitness and cognitive tests [14]. However, the impact of physical exercise on biochemical parameters in this patient population has not yet been evaluated. In this regard, serum levels of brain-derived neurotrophic factor (BDNF) may fluctuate depending on the level of exercise and may affect cognitive performance in patients with HGG. BDNF is a key molecule involved in brain plasticity, and it also plays an essential role in neurocognitive function [24,25]. Exercise has been shown to increase BDNF expression under both normal and pathological conditions [25]. However, the role of BDNF in HGG remains unclear. One study evaluated the association between BDNF and physical activity in HGG [26], finding that BDNF produced by glioblastoma-differentiated cells acts on glioblastoma stem cells, fostering their growth through paracrine signaling. A recent preliminary report suggested that BDNF, acting on different cells, can reorganize the brain microenvironment in such a way that it becomes resilient to neurodegeneration [24].

Proteins from the S100 family have been associated with the progression, diagnosis, and prognosis of glioma [27]. Changes in S100 protein levels induced by physical exercise have been studied in patients with multiple sclerosis [28] and in older people with vascular cognitive impairment [29]. However, studies on the relationship between the S100 protein and exercise in patients with HGG have not been undertaken.

Therefore, we hypothesized that training using augmented reality will have a beneficial effect on the physical and cognitive performance of patients with brain tumors treated with radiotherapy and will improve their quality of life. We also assume that exercise can have a positive impact on the selected blood parameters. In this context, we conducted a randomized clinical trial (RCT) to examine the effect of augmented-reality-based rehabilitation exercises on physical and functional fitness, cognitive function, fatigue, mood, QoL, selected blood parameters, BDNF, and S100 protein in patients with HGG.

## 2. Materials and Methods

### 2.1. Study Design

This was a randomized clinical trial involving patients scheduled to undergo radiotherapy after the surgical removal of a brain tumor. Patients were randomized to the active exercise group or to the control group. The study was conducted from October 2021 to April 2023 at the Radiotherapy Department at the Greater Poland Cancer Center in Poznan, Poland. The study protocol was approved by the local Bioethics Committee of the Poznan University of Medical Sciences (No. 703/18). The study was registered at clinicaltrials.gov (accessed on 9 September 2023) (identifier: NCT05192447) and was created as a result of the research project No. 2020/37/B/NZ7/01122 supported by the National Science Center.

### 2.2. Participants

The inclusion criteria were as follows: age 18–70 years; confirmed diagnosis of stage III or IV glioma (according to the 2021 World Health Organization Classification of Tumors of the CNS [30]); eligibility for radiotherapy; and good general physical condition (score of 0–2 on the Eastern Cooperative Oncology Group fitness scale). The exclusion criteria were: ≥2 brain lesions; psychological or psychiatric illness under pharmacological treatment; presence of other neurological disorders (e.g., multiple sclerosis, Parkinson’s disease); and/or significant clinical circulatory failure (New York Heart Association scale, stage III or IV).

Patients meeting the above criteria were given the opportunity to participate in the trial by their treating radiation oncologist. All participants were required to provide written informed consent to participate in the study. Participation was completely voluntary, and participants could withdraw at any time.

### 2.3. Sample Size

To determine the sample size, we performed a power analysis using G*Power [31] with the following assumptions: Cohen’s f for repeated measures ANOVA: 0.25; alpha: 0.05; power: 0.80; number of groups: 2; number of time points: 3. Based on these assumptions, the calculated minimum sample size was N = 44. To allow for dropouts, we recruited a total of 72 participants.

### 2.4. Radiotherapy Procedure

All patients received intensity-modulated radiotherapy (IMRT), delivered over 30 days under a conventional fractionation regimen (2 Gy per dose, total dose = 60 Gy) following the schedule described by Scaringi et al. [32].

### 2.5. Randomization

Patients were divided into the two study groups by simple randomization using a computer-generated list of random numbers.

### 2.6. Exercise Program

**Figure 1:**
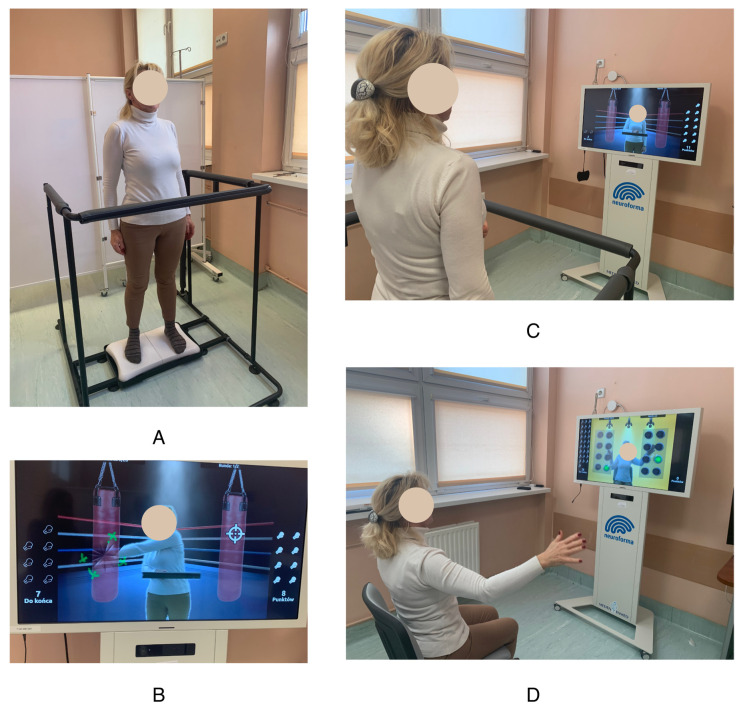
Exercises using augmented reality. (**A**) Standing position on the posturographic platform; (**B**,**C**) screen with the figure of the patient during exercise; (**D**) sitting position during the exercise.

Patients allocated to the exercise group undertook regular physical exercise according to an in-house protocol. The physical training schedule (duration, frequency, and intensity) was designed to meet the American Cancer Society’s recommendations for cancer patients [33]. Exercises were carried out at the hospital under the supervision of a qualified physiotherapist throughout the 30-day RT treatment period. Physical training was conducted before each RT session (23 h after the previous dose). Upon completion of the full 30-day RT program, the patients performed a set of exercises in the morning at home according to the same rules, using the Neuroforma remote program https://www.neuro-forma.com/science/ (accessed on 9 September 2023). Once a month, stationary in-person consultations with a physiotherapist were held in order to verify the effects and introduce any corrections to the training plan. Physical activity was moderate, with a maximum heart rate (HRmax) of 70% (as calculated by HRmax = 220−age) during training. Exercises were performed five times a week. The duration of the training session was 60 min, distributed as follows: 10 min of warm-up, 40 min of training, and 10 min of relaxation exercises. “Proper” training included 40 min of exercises using the Neuroforma neurorehabilitation device. This device allows users to perform exercise in an augmented reality environment using a posturographic platform with visual biofeedback. This device consists of a large display (20-inch monitor, which provides interaction feedback), a computer system, a 3D optical system enabling precise observation of patients’ activity, a balance platform for assessing balance, and a safety barrier. During the exercises, the patient stands or sits in front of the monitor screen. The camera system records his figure and movements. The exerciser sees a real, mirror image on the screen, with virtual objects appearing around it. The patient’s task is to direct his reflection in such a way as to catch, move, or hit the appearing objects. Figure 1 shows a patient performing exercises using an augmented reality device. The program allows users to choose from 20 games to improve the joint range of motion and muscle endurance of the upper limbs, eye–hand coordination, balance, reaction speed, and cognitive functions (including attention, memory, reading, and counting). During balance exercises, the patient stands on a wireless posturography platform. Static posturography is performed, recording the displacement of the center of gravity projection relative to the plane of the device platform by measuring the directions of foot pressure forces. During balance exercises, the patient sees an object on the screen, which he controls by balancing on the platform. Some of the exercises also require performing tasks with the upper limbs while controlling balance. In addition to the exercise function, monitoring of important posturographic parameters is also available: measurement of the ellipse area and center of pressure path. Using augmented reality technology, the patient receives immediate feedback on the correctness and level of exercise performance (i.e., biofeedback). For each exercise, the level of difficulty can be graduated as appropriate. The training was progressive. The physiotherapist selected the initial level of difficulty and increased or decreased it during the examination depending on the condition of the exerciser.

### 2.7. Control Group

The control group performed normal activities during the day. However, they were asked to record their physical activity using daily activity notes. Patients allocated to this group received the standard recommendations regarding the minimum level of physical activity [34].

### 2.8. Study Scheme

Study participants were examined at three different time points: (1) before the start of radiotherapy (T0), (2) the day after completion of the full RT program (T1), and (3) three months after completion of RT (T2).

### 2.9. Measurements

To assess the effectiveness of patients’ rehabilitation, questionnaires and research tools were used that are validated and widely used in the assessment of oncological patients, in particular patients with brain tumors. Both subjective and objective assessment tools were used. The Hand Grip Strength (HGS) test, 6-Minute Walk Test (6MWT), and Timed Up and Go (TUG) test are indicated as the most common cancer objective outcome measures [35].

A qualified physiotherapist conducted the fitness tests and assessed QoL and fatigue levels. A neuropsychologist was responsible for assessing cognitive function, depression, and anxiety. The following tests were administered.

#### 2.9.1. Hand Grip Strength (HGS) Test

The HGS is an indicator of overall muscle strength, physical fitness, and overall health and nutrition. HGS is a predictor of mortality and length of hospital stay [36]. This measurement is widely used to assess older individuals and cancer patients [37,38]. The HGS test was performed according to the guidelines established by the American Society of Hand Therapists [39] using a hand hydraulic dynamometer (JAMAR, Sammons Preston Rolyan, Bolingbrook, IL, USA). During this process, the participant was asked to sit in a chair without armrests or a backrest, with his/her feet located parallel on the floor. The knees and hips were flexed at 90 degrees. The arm of the tested hand was adducted to the trunk, the elbow was flexed at a right angle, the forearm was in a neutral position. Then, the participants were asked to grip a level with as much force as possible for 6 s. Three repetitions were performed, with a 1 min interval between them. The highest value achieved was used for the study.

#### 2.9.2. 6-Minute Walk Test (6MWT)

The 6MWT was used to assess functional capacity. This test is commonly used in clinical trials on cancer patients to estimate aerobic capacity [40]. The test was conducted following the guidelines of the American Thoracic Society [41]. A total distance of 30 m was divided into 3 m sections marked with tape on the floor. A 10 min rest was required before starting the test. During the test, the patient was asked to walk at a natural pace for 6 min. The parameter of interest was the total distance walked.

#### 2.9.3. Timed Up and Go (TUG) Test

The TUG test was used to assess functional mobility. This is a reliable, validated tool originally developed to assess mobility in older populations, but it is also widely used for younger populations. It is used to measure response to treatment in terms of improvement in function and QoL. During the test, the patient sits in a chair, with his/her back against the backrest with the forearms on the armrests. The patient is then asked to stand up and walk a total of 3 m (marked with tape on the floor) at normal speed. The patient then turns around, returns to the chair, and sits down. The task was performed three times, and the times were averaged for the statistical analysis [42].

#### 2.9.4. The Functional Independence Measure (FIM)

The FIM is an 18-item scale recommended for use on patients with neurological illnesses. This scale is designed to evaluate physical, psychological, and social function [43]. It is also used in the evaluation of oncological patients with both primary and metastatic brain tumors [44]. It assesses performance in six areas: self-care, continence, mobility, transfers, communication, and cognition [43]. The scale assesses the patient’s degree of dependence on the help of others in everyday activities. This tool is used to assess a patient’s level of disability and changes in response to rehabilitation or a medical intervention [43,44].

#### 2.9.5. Quality of Life

The Polish language version of the FACT-Br (Functional Cancer Therapy Assessment—Brain) questionnaire, originally developed for use in clinical trials, was used to assess QoL [45]. This questionnaire was developed specifically for the assessment of patients with primary brain tumors and is widely used for this purpose [46]. This scale contains 51 questions. The scale is considered highly accurate, has good psychometric properties, and is an effective measure of QoL in patients with brain tumors. The instrument includes physical, functional, familial, social, and emotional domains and other items specific to the problems commonly encountered by patients with brain tumors. Respondents answer questions on a five-point Likert scale, ranging from “0” (“not at all”) to “4” (“very much”) [47].

#### 2.9.6. Fatigue

The Functional Assessment of Chronic Illness Therapy—Fatigue (FACIT-F) scale was used to assess symptoms of fatigue. This tool is widely used to assess fatigue in patients with cancer and has good internal consistency and test–retest reliability. This scale consists of 13 items to measure an individual’s level of fatigue during ADLs over the past week. Responses are given on a 5-point Likert scale and range from 0 to 4. Total scores range from 0 to 52, with higher scores indicating less fatigue. Scores < 30 are considered to indicate severe fatigue [48].

#### 2.9.7. Depression and Anxiety

The Hospital Anxiety and Depression Scale (HADS) was used to assess depressive and anxiety symptoms. This self-report questionnaire is commonly used to assess emotional stress, including for cancer patients. HADS consists of two subscales to evaluate anxiety and depression over the past week. Each subscale consists of seven items with four possible response options. Total scores on each subscale range from 0 to 21, with higher total scores indicating greater levels of anxiety or depression [49].

#### 2.9.8. Addenbrooke’s Cognitive Examination III (ACE III)

ACE III was used to assess cognitive function. This screening tool can differentiate between participants with and without cognitive impairment. The questionnaire consists of 21 questions divided into five sections (attention, memory, fluency, language, and visuospatial processing). The maximum test score is 100, which should be interpreted in the context of the patient’s overall history and clinical examination [50]. ACE III shows its accuracy in assessing cancer patients and is indicated as an important tool for quick and easy neurocognitive function assessment in patients with glioma [51].

#### 2.9.9. Laboratory Tests

Blood was drawn via a venous puncture after overnight fasting. Patients were asked to avoid intense physical activity in the 24 h before blood sampling. The serum and plasma samples were stored at −80 °C until final analysis.

Venous blood samples were taken to measure hemoglobin (Hb), white blood cells, red blood cells, neutrophils, lymphocytes, and monocytes. Platelets were also obtained and processed by a centralized laboratory. Biochemical markers were measured using the Cobas 6000TM clinical chemistry analyzer (Roche; Mannheim, Germany). Hematological indices (complete blood count, hemoglobin) were analyzed in EDTA-blood with the XT-2000iTM (Sysmex Corporation; Kobe, Japan).

We also measured alanine aminotransferase (ALT), aspartate aminotransferase (AST), creatine kinase (CK), sodium, creatinine, and bilirubin. These parameters were determined quantitatively in serum using the COBAS 6000 analyzer. AST, CK, and CKMB tests were performed using kinetic methods with absorbance measurement. High-sensitivity C-reactive protein (hsCRP) was determined using an immunoturbidimetric method enhanced with latex particles.

BDNF was evaluated in sera and in peripheral blood mononuclear cell lysates using the enzyme-linked immunoassay (ELISA) method. The astrocytic protein S-100 was estimated using ELISA. To improve the quality of the results and to shorten the reading time, we used the Synergy HTX system to automate the reading of ELISA tests.

### 2.10. Statistical Analysis

Statistical analysis was performed using the Statistica 13.3 software (TIBCO Software, Poland). The threshold for statistical significance was set at *p* < 0.05. The Shapiro–Wilk test was used to check distribution normality. The ANOVA test for repeated measures was used to examine differences over time. The assumption of sphericity was checked using the Mauchley test; if sphericity was violated, the Greenhaus–Geisser correction was applied. Tukey’s test was used for post hoc analyses. For variables with a non-normal distribution, and in comparisons of ordinal variables, we applied Friedman’s test with Dunn’s post-hoc test. Student’s t test was used for the comparison of groups with normally distributed variables; the Mann–Whitney test was used to compare groups with a non-normal distribution and for ordinal variables.

## 3. Results

**Figure 2:**
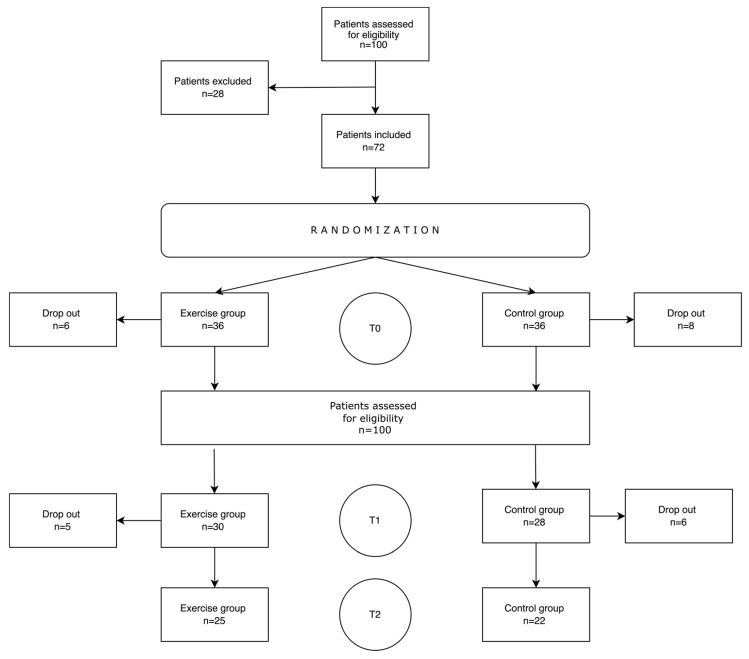
Flow of participants through the study.

A total of 100 patients were assessed for study eligibility. Of these, 28 were excluded for the following reasons: lack of consent (*n* = 13); age > 70 years (*n* = 4); more than two tumors (*n* = 4); neurological disorders (*n* = 3); NYHA III or IV (*n* = 3). During the first assessment (T0), a total of fourteen patients (six in the experimental group and eight in the controls) withdrew consent. At the second assessment (T1), a recurrence was detected in seven patients (three in the experimental group and four in controls), one patient in the exercise group died, and six patients (two and four in the exercise and control groups, respectively) withdrew. Figure 2 shows the study flow diagram.

Overall, the mean age of the study participants was 52.58 ± 14.21 years. Patients in the exercise group were, on average, significantly younger than controls (Table 1). For this reason, we initially compared the two groups in terms of independence on the FIM scale and cognitive functioning, finding no differences. Most patients in both groups were men. All participants underwent surgical resection, and all received radiotherapy and chemotherapy.

The characteristics of the participants according to treatment allocation are presented in Table 1.

### 3.1. Physical Fitness, Mental Health, and Quality of Life Results

In the controls, we observed a statistically significant decrease in HGS between T0 and T2 (*p* = 0.017). By contrast, HGS values in the experimental group increased, although not significantly. Table 2 and Table 3 show changes in results over time by group. No significant changes in the results of the other fitness tests were observed in either group. QoL decreased in both groups between T0 and T2, but this change was not statistically significant.

The ACE III domain attention score decreased significantly in the controls (*p* = 0.047) but remained unchanged in the exercise group.

Table 4 shows the individual measurements of the two groups (Table 4). There were statistically significant between-group differences in changes in HGS between T0 and T1 (*p* = 0.015) and between T0 and T2 (*p* = 0.006). In the experimental group, HGS increased by 2.59 ± 5.14 kg between T0 and T1 and by 2.91 ± 4.87 kg between T0 and T2. By contrast, in the control group, these values decreased by 1.82 ± 2.75 kg and 8.00 ± 9.54 kg, respectively. The changes in the time taken to perform the TUG test between the T0 and T2 were statistically significantly in both groups (*p* = 0.029), decreasing by 0.02 ± 1.08 s in the exercise group and increasing by 1.75 ± 2.31 s in the control group.

Significant differences were observed between the groups regarding the change in the HADS depression results between T0 and T1(*p* = 0.022), with an increase of 2.09 ± 5.86 points in the control group and a decrease of 1.69 ± 3.42 in the experimental group.

**Figure 3:**
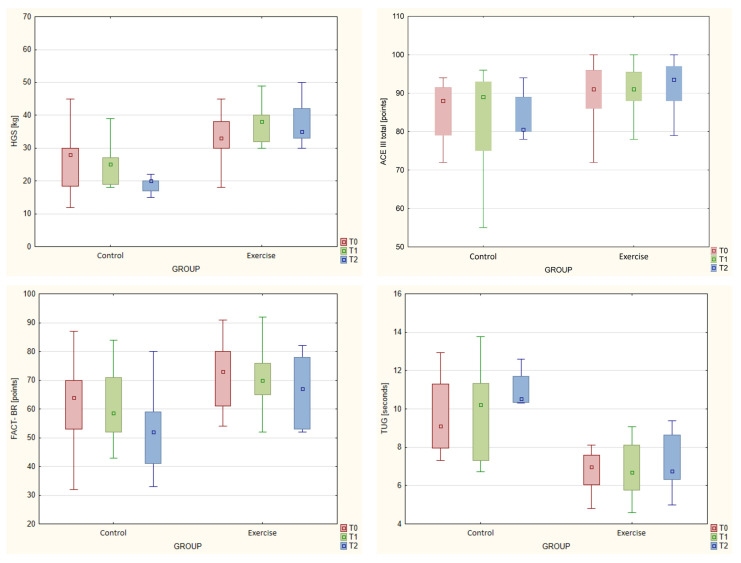
Box and whisker charts showing changes over time in selected variables across the two groups.

Selected results are presented in Figure 3.

### 3.2. Laboratory Test Results

Regarding the laboratory tests, statistically significant changes were only observed for mean corpuscular volume (MCV) and mean corpuscular hemoglobin (MCH), with significant increases in both the experimental (MCV, *p* = 0.029 and MCH, *p* = 0.023) and control group (*p* = 0.014 and *p* = 0.029, respectively). Bilirubin levels increased by 0.30 ± 0.41 in the experimental group and decreased by 0.07 ± 0.10 mg/dL in the control group, presenting a significant between-group difference (*p* = 0.019). Detailed laboratory test results for both groups are presented in the Appendix (Table A1, Table A2 and Table A3).

## 4. Discussion

The main aim of this RCT was to determine the effects of an augmented-reality-based physical and cognitive rehabilitation program on physical and cognitive function, mental health, laboratory parameters, and QoL in patients with HGG undergoing radiotherapy. Our main finding was that the exercise group experienced no significant changes in any of the parameters. By contrast, the controls experienced a significant decrease in hand grip strength and a decline in attention (ACE III). These findings suggest that exercise during radiation therapy may prevent loss of muscle strength and attention in this patient population.

Much is already known about the positive impact of physical exercise on oncological patients. Nearly 700 clinical trials involving more than 50,000 cancer patients have demonstrated the positive effects of exercise during the treatment and recovery phases, especially in ameliorating side effects such as fatigue, mental stress, and physical limitations [52]. However, in patients with HGG, less attention has been paid to physical and cognitive rehabilitation. Studies have shown that most patients with a primary brain tumor are open to exercise suggestions and that most patients are able to participate in an exercise program during and after cancer treatment [53,54]. Our research was one of the few studies that used augmented reality in the exercise program. It is worth noting that the study used non-wearable equipment that did not employ full immersion. In previous studies in other patient groups, head-mounted display caused some patients to experience nausea and headaches [55].

At present, there is no standardized exercise protocol for patients with brain glioma. A mini review published in 2023 [10] found that the most successful interventions included personalized exercise recommendations, individualized training, and adherence strategies such as training data monitoring and regular guidance from a physiotherapist. Based on the findings of that review, we decided to offer patients augmented reality training because it allows the physiotherapists to personalize and regularly monitor the exercise program, both during the stay in the ward and at home.

One of the key aims of our study was to assess the impact of exercise on physical fitness (measured by the HGS, TUG, and 6MWT tests). Previous studies on patients with low-grade glioma have found reduced muscle strength and cardiorespiratory capacity during cancer treatment [56]. For this reason, we wanted to see if exercise could prevent this process in HGG patients. Based on the result of the HSG test—in which the exercise group maintained their strength level while the controls lost some strength—it seems clear that exercise can help patients maintain their strength over time. In terms of functional mobility, the results of the TUG tests also suggest the benefit of exercise, as evidenced by the significant between-group differences in the TUG test results between T0 and T2 (a 1.75 s increase in controls vs. no change in the exercise group). Regarding the 6MWT, there were no significant changes over time in either group, and no significant differences in mean scores between the groups. Nowak et al. [57] recently evaluated the impact of resistance and aerobic training on HGG patients during radiochemotherapy. Those researchers observed a significant increase in lower limb muscle strength before and after the intervention (6 weeks). Unfortunately, that study did not include a control group. Eisenhut et al. [58] compared the effect of two types of physical training (endurance and strength) on the physical fitness of patients with glioma (WHO grades III and IV). Interestingly, while muscle strength (measured by HGS) improved in both groups, 6MWT scores improved only in the active control group (but not in the endurance training group). Given the findings described above, it seems clear that more research is needed to better determine the effects of different types of training on muscle strength during oncological treatment in patients with glioblastoma. Our findings suggest that while exercise may not significantly improve strength, it could prevent a decline during and after oncological treatment, which would have a direct positive impact on the functional efficiency of these patients.

The only significant change regarding the cognitive tests was a decrease in the attention domain of the ACE III test in the control group. Importantly, attention did not decrease in the exercise group, suggesting that exercise may be preventative. In the control group, we observed a decrease in mean total ACE III scores, but this was not statistically significant. Attention is one of the cognitive domains most often disturbed in patients with glioma [59]. However, we have not found any studies that have assessed the impact of physical exercise on cognitive function in patients with HGG during radiochemotherapy using validated neuropsychological tests. Gehring et al. [60] found that aerobic exercise had a positive impact on cognitive function, but this study was performed on patients who had completed oncological treatment at least 6 months before enrolment. Similarly, in other types of cancer, the relationship between exercise and cognitive function during cancer treatment is not well understood. A national cohort study (in the United States) of 580 breast cancer patients and 363 age-matched controls showed that patients who met the physical activity guidelines (defined as 150 min per week of moderate-to-vigorous physical activity) during chemotherapy had significantly better cognitive function than those who did not meet the recommended amount of exercise [61]. A protocol was recently published describing a new study [62] to determine the impact of intensive interval training on cognitive function in breast cancer patients. That study will undoubtedly shed more light on this topic when the results are published.

The positive benefits of physical activity on cognitive function are generally believed to be attributable to the exercise-induced expression of neurotrophic and neuroprotective markers (including BDNF), which promote neurogenesis in certain areas of the brain [63]. This is important given that chemoradiation may contribute to hippocampal degeneration [64,65] while exercise has been shown to improve hippocampal-dependent cognition and to increase hippocampal volume [66,67,68]. Exercise also reduces inflammatory markers, which are commonly elevated in response to aggressive cancer treatment [69]. To our knowledge, our study is the first to determine changes in BDNF levels in response to training in patients with HGG during oncological treatment. However, we observed no significant changes in BDNF levels over time in either group, suggesting that exercise may not impact BDNF, a finding that is consistent with a study by Miklja et al. [26], who found that the level of exercise in adults with glioblastoma had no effect on the circulating secretion of BDNF. Nonetheless, those findings must be interpreted cautiously given that physical activity was self-reported in that study. In a study involving women with ovarian cancer [70], Cartmel et al. found no differences in BDNF levels between exercisers and non-exercisers. By contrast, a meta-analysis of randomized clinical trials found that exercise increases BDNF concentration in plasma in healthy people [71]. Similarly, one study found that exercise increased BDNF in patients with neurodegenerative disorders [72]. However, the impact of physical activity on BDNF levels in patients with cancer (including HGG), remains unclear at present. In this regard, we believe that the study by Miklja et al. [26], together with our study, may serve as a basis for further research into this question.

We found no significant changes over time in either group in terms of QoL or fatigue levels, nor did we observe any between-group differences in these variables. In both groups, QoL worsened over time, but not significantly. In the control group, the level of fatigue was similar at all three time points. By contrast, perceived fatigue increased in the experimental group, but not significantly. Nowak et al. examined the effects of physical training on patients with glioblastoma [57], finding no improvement in fatigue or QoL, but—importantly—no deterioration in either of these variables over time. Eisenhut et al. [58] carried out of a similar study, which showed an increase in fatigue in the exercise groups and a decrease in the active control group. Hansen et al. also found that physiotherapy based on an exercise intervention and occupational therapy did not positively impact HRQOL [12]. By contrast, other researchers have found that exercise has a positive impact on fatigue and QoL [73]. The findings of the aforementioned studies suggest that, unlike other types of cancer [74,75], it may be more difficult to alleviate fatigue in patients with glioblastoma; alternatively, perhaps the dose and type of training were not appropriate. In any case, it is clear that more research is warranted.

Although we found no significant changes in either group in anxiety or depression, the control group presented worse HADS depression scores between T0 and T1 compared to the exercise group (Table 4). This finding suggests that physical exercise during radiochemotherapy may help to prevent depression during hospitalization. Other researchers [58] found that the endurance training and control groups had a positive change in depressive symptoms while the strength training group did not.

To our knowledge, this is the first study to examine the effects of exercise on blood chemistry and the permeability of the blood–brain barrier (BBB) (S100 protein) in patients with HGG during radiotherapy. However, we found no statistically significant changes in S100 protein levels in either group and no differences between the groups. In controls, S100 levels decreased slightly, while these levels remained unchanged in the exercise group. In studies involving older women, physical exercise appears to seal the BBB [76]. However, this effect was not observed in our sample of patients with HGG.

Bilirubin levels increased in the exercise group but decreased in controls. This finding may or may not be relevant given that many factors, including chemotherapy, can influence bilirubin levels. However, this finding is consistent with other studies showing that physical activity increases plasma bilirubin levels [77], which can cause certain adaptations and beneficial metabolic changes in people who exercise [78].

This study has several limitations. First, the study design did not consider the interaction between the studied variables. Further research focused on this topic should be based on multivariate analyses. Another study limitation is that we did not directly assess the level of physical activity in the control group, but rather we relied on the patients’ self-reported declaration of daily activity. By contrast, an important strength of our study is that it is the first to determine and compare BDNF and S100 protein levels in exercising and non-exercising HGG patients during radiotherapy. Another strength is that we measured a large range of parameters at three distinct time points (pre- and post-RT, and at 3 months of follow-up), thus providing a clear picture of how these parameters change over time. These novel data complement the findings of other studies, thus broadening our understanding of the effects of exercise in this patient population.

## 5. Conclusions

In conclusion, the results of this trial suggest that augmented reality training in HGG patients during and after radiotherapy can prevent the decline of muscle strength and attention. The effects of physical training on blood parameters, BDNF, and S100 protein levels remain unclear.

## Figures and Tables

**Table 1:** Characteristics of the study groups.

Variable (Mean ± SD or Median (Range) or *n* (%))	Exercise Group, *n* = 25	Control Group, *n* = 22	*p*-Value
Age, years	45.59 ± 11.15	60 ± 13.55	0.002
Sex			0.1
Female	3 (17.65)	7 (43.75)	
Male	14 (82.35)	9 (56.25)	
Time from tumor resection, weeks	4.38	4.29	0.7
Education			0.03
Primary	0	0	
Vocational	2 (11.76)	5 (31.25)	
Secondary	7 (41.18)	10 (62.50)	
High	8 (47.06)	1 (6.25)	
FIM	124.5 (121–126)	122 (86–126)	0.083
ACE III	91 (72–100)	88 (60–94)	0.052

Abbreviations: ACE III, Addenbrooke’s Cognitive Examination III; FIM, The Functional Independence Measure; SD, standard deviation.

**Table 2:** Physical fitness, mental health, and quality of life results at the three study time points in the exercise group.

Parameters (Mean ± SD or Median [Range])	Baseline	After RT	After 3 Months	*p*-Value
HGS, kg	32.76 ± 8.24	35.35 ± 9.59	35.73 ± 9.21	0.092
TUG, s	7.25 ± 2.03	7.22 ± 1.90	7.55 ± 2.48	0.695
6MWT, m	483.59 ± 116.48	500.74 ± 114.90	473.45 ± 85.20	0.385
FIM self-care	42 (42–42)	42 (42–42)	42 (12–42)	0.135
FIM sphincter control	14 (14–14)	14 (14–14)	14 (10–14)	0.368
FIM transverse	21 (21–21)	21 (21–21)	21 (9–21)	0.135
FIM locomotion	14 (13–14)	14 (12–14)	14 (6–14)	0.038
FIM communication	14 (12–14)	14 (12–14)	14 (13–14)	0.607
FIM social cognition	20 (16–21)	20 (15–21)	20 (16–21)	0.482
FIM total score	124.5 (121–126)	125 (119–126)	124 (69–126)	0.575
FACT-G PWB	22 (18–28)	23 (19–28)	21.5 (5–25)	0.509
FACT-G SWB	23 (13–27)	23 (16–28)	21 (13–28)	0.273
FACT-G EWB	18 (7–24)	18.5 (7–24)	16 (6–20)	0.84
FACT-G FWB	21 (10–28)	20.5 (10–28)	16 (0–24)	0.067
FACT-G total	80 (66–107)	82.5 (57–108)	76.0 (47–95)	0.093
FACT-BR	73 (54–91)	70 (52–92)	67 (52–82)	0.074
FACIT-F	42 (28–52)	40 (28–52)	33 (22–49)	0.068
ACE III attention	18 (8–18)	18 (15–18)	18 (13–18)	0.951
ACE III memory	23 (12–26)	24 (10–26)	23.5 (11–25)	0.421
ACE III fluency	11 (1–14)	10.5 (6–14)	11.5 (2–20)	0.664
ACE III language	26 (18–26)	26 (22–26)	26 (21–26)	0.074
ACE III VS	16 (15–16)	16 (13–16)	15.5 (8–15)	0.485
ACE III total	91 (72–100)	91 (78–100)	93.5 (59–100)	0.749
HADS anxiety	3 (0–14)	0 (0–18)	0 (0–5)	0.125
HADS depression	2 (0–9)	0 (0–7)	0 (0–6)	0.146

Abbreviations: RT, radiotherapy; SD, standard deviation; HGS, hand grip strength; TUG, Time Up and Go test; 6MWT, six-minute walk test; FIM, The Functional Independence Measure; FACT-G, Functional Cancer Therapy Assessment—General; PWB, physical wellbeing; SWB, social wellbeing; EWB, emotional wellbeing; FWB, functional wellbeing; FACT-BR, Functional Cancer Therapy Assessment—Brain; FACIT-F, The Functional Assessment of Chronic Illness Therapy—Fatigue; ACE III, Addenbrooke’s Cognitive Examination III; VS, visuospatial; HADS, The Hospital Anxiety and Depression Scale.

**Table 3:** Physical fitness, mental health, and quality of life results at the three study time points in the control group.

Parameters (Mean ± SD or Median (Range)	Baseline	After RT	After 3 Months	*p*-Value
HGS, kg	26.63 ± 12.08	26.55 ± 10.65	17.89 ± 4.23	0.017 ^b^
TUG, s	10.03 ± 3.03	9.63 ± 2.36	10.75 ± 1.33	0.197
6MWT, m	382.21 ± 68.52	379.91 ± 85.65	361 ± 72.07	0.375
FIM self-care	42 (30–42)	42 (42–42)	42 (18–42)	0.156
FIM sphincter control	14 (8–14)	14 (14–14)	14 (12–14)	0.368
FIM transverse	21 (20–21)	21 (21–21)	21 (9–21)	0.368
FIM locomotion	14 (10–14)	14 (14–14)	14 (6–14)	0.368
FIM communication	14 (7–14)	14 (12–14)	14 (11–14)	0.368
FIM social cognition	18.5(11–21)	21 (16–21)	18.5 (10–21)	0.670
FIM total score	12 (86–126)	126 (121–126)	121.5 (68–126)	0.368
FACT-G PWB	22 (10–28)	22 (10–28)	15 (4–22)	0.089
FACT-G SWB	24.5 (20–28)	23 (4–28)	20.5 (2–28)	0.326
FACT-G EWB	16 (0–24)	15 (11–24)	13.5 (3–18)	0.698
FACT-G FWB	16.5(2–28)	17 (6–27)	18 (7–22)	0.738
FACT-G total	79.5 (41–100)	88 (43–101)	71 (18–83)	0.648
FACT-BR	64 (32–87)	58.5 (43–84)	52 (33–80)	0.085
FACIT-F	36.5 (11–52)	40 (20–50)	36.5 (3–49)	0.379
ACE III attention	18 (12–18)	18 (13–18)	17.5 (11–18)	0.047
ACE III memory	20 (12–26)	21 (9–26)	19.5 (10–26)	0.972
ACE III fluency	9.5 (1–13)	9.5 (1–14)	8.5 (1–12)	0.167
ACE III language	25 (17–26)	25.5 (17–26)	25 (20–26)	0.834
ACE III VS	15 (8–17)	15.5 (6–17)	15.5 (8–16)	0.350
ACE III total	88 (60–94)	89 (55–96)	80.5 (55–94)	0.558
HADS anxiety	4 (0–12)	4 (0–7)	7 (0–11)	0.639
HADS depression	3 (0–20)	4 (0–15)	3(0–9)	0.507

^b^. difference in post-hoc test between baseline and after 3 months. Abbreviations: RT, radiotherapy; SD, standard deviation; HGS, hand grip strength; TUG, Time Up and Go test; 6MWT, six-minute walk test; FIM, The Functional Independence Measure; FACT-G, Functional Cancer Therapy Assessment—General; PWB, physical wellbeing; SWB, social wellbeing; EWB, emotional wellbeing; FWB, functional wellbeing; FACT-BR, Functional Cancer Therapy Assessment—Brain; FACIT-F, The Functional Assessment of Chronic Illness Therapy—Fatigue; ACE III, Addenbrooke’s Cognitive Examination III; VS, visuospatial; HADS, The Hospital Anxiety and Depression Scale.

**Table 4:** Mean differences between measurements of physical fitness, mental health, and quality of life at different time points for the exercise and control groups.

Parameters	Difference between T_0_ and T_1_		Difference between T_1_ and T_2_		Difference between T_0_ and T_2_	
	EG	CG	EG	CG	EG	CG
HGS, kg	2.59 ± 5.14 *	−1.82 ± 2.75 *	−0.45 ± 6.46	−6.33 ± 7.33	2.91 ± 4.87 *	−8.00 ± 9.54 *
TUG, s	−0.03 ± 073	0.29 ± 1.61	−0.02 ± 0.95	1.68 ± 1.6	−0.02 ± 1.08 *	1.75 ± 2.31 *
6MWT, m	16.88 ± 33.16	−8.73 ± 54.17	−3.55 ± 54.69	−30.71 ± 41.89	16.45 ± 68.13	−21.86 ± 65.88
FIM total score	−1.44 ± 2.06	0.36 ± 2.3	−6.77 ± 16.16	−7.50 ± 20.42	−8.23 ± 16.31	−7.44 ± 19.22
FACT-G PWB	0.44 ± 1.67	−0.73 ± 3.26	−3.09 ± 5.2	−4.43 ± 5.44	−2.83 ± 5.1	−2.5 ± 5.11
FACT-G SWB	−1.21 ± 3.28	−2.73 ± 7.09	−0.82 ± 2.93	−0.14 ± 2.34	−1.25 ± 3.89	−3.75 ± 7.11
FACT-G EWB	−0.19 ± 2.9	0.18 ± 3.74	−1.36 ± 5.03	−1.00 ± 3.11	−1.50 ± 4.32	−2.00 ± 4.96
FACT-G FWB	0.56 ± 5.4	0.00 ± 6.07	−3.55 ± 5.91	−0.57 ± 7.04	−3.75 ± 6.17	−1.25 ± 6.73
FACT-G total	−0.50 ± 8.47	−3.27 ± 9.25	−5.18± 14.65	−6.14 ± 9.15	−7.27 ± 8.34	−14.38 ± 22.18
FACT-BR	−2.29 ± 5.57	−2.25 ± 10.45	−4.0 ± 11.1	−6.00 ± 7.00	−7.00 ± 11.23	−7.00± 9.93
FACIT-F	−2.53 ± 10.9	1.09 ± 6.19	−2.58 ± 9.77	−4.00 ± 15.81	−6.66 ± 8.18	−2.30 ± 13.02
ACE III attention	0.31 ± 2.52	−0.29 ± 0.73	−0.15 ± 069	−0.90 ± 1.85	−0.43 ± 1.65	−1.00 ± 2.16
ACE III memory	0.81 ± 3.29	0.14 ± 3.16	−2.23 ± 4.3	−0.10 ± 2.08	−1.00 ± 5.45	0.20 ± 3.74
ACE III fluency	0.19 ± 2.59	−0.07 ± 1.64	0.69 ± 4.94	−1.40 ± 2.22	0.71 ± 5.62	−1.00 ± 1.7
ACE III language	0.06 ± 1.18	−0.86 ± 2.03	−0.54 ± 1.45	0.10 ± 3.14	−0.57 ± 1.34	−0.10 ± 3.31
ACE III VS	−0.31 ± 1.01	−0.21 ± 1.31	−0.46 ± 1.76	−0.80 ± 1.93	−1.00 ± 0.29	−0.70 ± 2.0
ACE III total	1.19 ± 5.21	−1.50 ± 7.53	−2.85 ± 9.58	−3.20 ± 6.55	−2.29 ± 12.03	−2.50 ± 7.49
HADS anxiety	−2.13 ± 4.49	−0.27 ± 3.35	−1.38 ± 5.35	2.13 ± 3.64	−2.62 ± 4.41	0.22 ± 6.1
HADS depression	−1.69 ± 3.42 *	2.09 ± 5.86 *	−0.46 ± 1.9	−2.13 ± 4.52	−1.69 ± 3.25	−2.22 ± 7.38

* *p* < 0.05. Abbreviations: EG, exercise group; CG, control group; HGS, hand grip strength; TUG, Time Up and Go test; 6MWT, six-minute walk test; FIM, The Functional Independence Measure; FACT-G, Functional Cancer Therapy Assessment—General; PWB, physical wellbeing; SWB, social wellbeing; EWB, emotional wellbeing; FWB, functional wellbeing; FACT-BR, Functional Cancer Therapy Assessment—Brain; FACIT-F, The Functional Assessment of Chronic Illness Therapy—Fatigue; ACE III, Addenbrooke’s Cognitive Examination III; VS, visuospatial; HADS, The Hospital Anxiety and Depression Scale.

## Data Availability

The datasets generated for this study are available upon request to the corresponding author.

## Appendix A

**Table A1:** Laboratory results at three time points in the exercise group.

Parameters (Mean ± SD)	Baseline	After RT	After 3 Months	*p*-Value
S-100 µg/L	0.04 ± 0.02	0.04 ± 0.02	0.04 ± 0.02	0.223
Sodium mmol/L	139.88 ± 2.06	142.63 ± 4.37	139.17 ± 2.48	0.459
Potassium mmol/L	4.25 ± 0.36	4.35 ± 0.57	4.00 ± 0.15	0.789
Glucose mg/dL	94.69 ± 7.85	95.45 ± 15.58	99.50 ± 14.34	0.534
Creatinine mg/dL	0.87 ± 0.17	0.92 ± 0.18	0.97± 0.22	0.496
AST U/L	18.44 ± 5.34	18.90 ± 9.61	20.83 ± 4.36	0.311
ALT U/L	26.69 ± 12.20	34.80 ± 32.50	33.67± 18.77	0.401
Bilirubin mg/dL	0.50 ± 0.37	0.66 ± 0.41	0.76 ± 0.50	0.105
WBC G/L	7.95 ± 2.46	8.25 ± 3.54	8.55 ± 3.76	0.937
LYM G/L	2.01 ± 0.67	1.79 ± 0.90	1.73 ± 0.84	0.148
NEU G/L	5.14 ± 1.89	5.69 ± 2.93	5.97 ± 3.35	0.913
MON G/L	0.60 ± 0.26	0.62 ± 0.27	0.65 ± 0.26	0.803
EOS G/L	0.12 ± 0.10	0.14 ± 0.17	0.18 ± 0.25	0.661
IG G/L	0.08 ± 0.12	0.07 ± 0.07	0.12 ± 0.20	0.704
BASO G/L	0.04 ± 0.03	0.01 ± 0.02	0.02 ± 0.02	0.423
RBC T/L	4.71 ± 0.38	4.67 ± 0.56	4.68 ± 0.53	0.523
HBG mmol/L	8.59 ± 0.74	8.85 ± 1.07	8.69 ± 0.71	0.289
HCT L/L	0.42 ± 0.03	0.43 ± 0.05	0.42 ± 0.03	0.307
MCV fL	88.82 ± 4.89	91.34 ± 4.15	90.98 ± 7.24	0.029 ^b^
MCH fmol	1.83 ± 0.13	1.90 ± 0.10	1.88 ± 0.19	0.023 ^b^
MCHC mmol/L	20.54 ± 0.55	20.79 ± 0.43	20.57 ± 0.53	0.305
PLT G/L	267.94 ± 81.01	187.60 ± 51.45	229.17 ± 55.62	0.148
BDNF pg/mL	233.89 ± 222.25	242.94 ± 188.68	330.67 ± 297.57	0.847

^b^. difference in post-hoc test between baseline and after 3 months. Abbreviations: RT, radiotherapy; SD, standard deviation; AST, aspartate aminotransferase; ALT, alanine aminotransferase; WBC, white blood count; LYM, lymphocytes; NEU, neutrophils; MON, monocytes; EOS, eosinophils; IG, immunoglobulins; BASO, basophils; RBC; red blood cell; HBG; hemoglobin; HCT, hematocrit; MCV, mean corpuscular volume; MCH, mean corpuscular hemoglobin; MCHC, mean corpuscular hemoglobin concentration; PLT, platelet count; BDNF, brain-derived neurotrophic factor.

**Table A2:** Laboratory results at three time points in the control group.

Parameters (Mean ± SD)	Baseline	After RT	After 3 Months	*p*-Value
S-100 µg/L	0.13 ± 0.17	0.06 ± 0.05	0.07 ± 0.04	0.105
Sodium mmol/L	139.50 ± 2.99	139.17 ± 3.06	140.25 ± 2.19	0.09
Potassium mmol/L	4.12 ± 0.22	4.05 ± 0.33	4.07 ± 0.22	0.441
Glucose mg/dL	110.63 ± 27.50	98.86 ± 26.28	108.88 ± 26.71	0.779
Creatinine mg/dL	0.79 ± 0.10	0.87 ± 0.15	0.77 ± 0.05	0.075
AST U/L	18.93 ± 5.93	23.33 ± 16.00	20.00 ± 9.01	0.738
ALT U/L	31.53 ± 23.51	43.44 ± 17.52	39.75 ± 29.60	0.513
Bilirubin mg/dL	0.39 ± 0.12	0.44 ± 0.12	0.30 ± 0.09	0.094
WBC G/L	8.76 ± 3.88	6.90 ± 2.01	6.83 ± 2.60	0.399
LYM G/L	1.71 ± 0.76	1.34 ± 0.54	1.23 ± 0.65	0.186
NEU G/L	6.34 ± 3.32	5.14 ± 1.72	5.22 ± 2.36	0.686
MON G/L	0.56 ± 0.23	0.53 ± 0.21	0.38 ± 0.14	0.206
EOS G/L	0.07 ± 0.07	0.07 ± 0.11	0.04 ± 0.06	0.513
IG G/L	0.14 ± 0.24	0.06 ± 0.10	0.11 ± 0.18	0.216
BASO G/L	0.03 ± 0.04	0.01 ± 0.01	0.02 ± 0.03	0.129
RBC T/L	4.34 ± 0.40	4.30 ± 0.36	4.08 ± 0.43	0.149
HBG mmol/L	8.31 ± 0.77	8.34 ± 0.71	8.08 ± 0.80	0.658
HCT L/L	0.40 ± 0.03	0.40 ± 0.04	0.39 ± 0.03	0.760
MCV fL	92.31 ± 3.05	93.26 ± 2.31	96.37 ± 5.64	0.014 ^b^
MCH fmol	1.91 ± 0.07	1.94 ± 0.06	1.99 ± 0.10	0.0285 ^a,c^
MCHC mmol/L	20.75 ± 0.47	20.84 ± 0.45	20.65 ± 0.45	0.559
PLT G/L	253.75 ± 77.37	211.86 ± 71.76	187.10 ± 52.20	0.202
BDNF pg/mL	386.02 ± 369.26	227.69 ± 149.58	143.24 ± 99.94	0.234

^a^. difference in post-hoc test between baseline and after RT; ^b^. difference in post-hoc test between baseline and after 3 months; ^c^. difference in post-hoc test between after RT and after 3 months. Abbreviations: RT, radiotherapy; SD, standard deviation; AST, aspartate aminotransferase; ALT, alanine aminotransferase; WBC, white blood count; LYM, lymphocytes; NEU, neutrophils; MON, monocytes; EOS, eosinophils; IG, immunoglobulins; BASO, basophils; RBC; red blood cell; HBG; hemoglobin; HCT, hematocrit; MCV, mean corpuscular volume; MCH, mean corpuscular hemoglobin; MCHC, mean corpuscular hemoglobin concentration; PLT, platelet count; BDNF, brain-derived neurotrophic factor.

**Table A3:** Mean differences between laboratory tests in both groups at three time points.

Parameters	T0 and T1		T1 and T2		T0 and T2	
	EG	CG	EG	CG	EG	CG
S-100 µg/L	0.00 ± 0.02	−0.07 ± 0.17	−0.01 ± 0.02	−0.01 ± 0.07	−0.01 ± 0.02	−0.11 ± 0.19
Sodium mmol/L	2.63 ± 4.60	0.17 ± 2.48	−3.50 ± 7.90	1.80 ± 1.10	0.00 ± 3.10	1.75 ± 2.25
Potassium mmol/L	0.18 ± 0.67	−0.04 ± 0.33	0.16 ± 0.15	0.10 ± 0.29	−0.01 ± 0.35	−0.06 ± 0.28
Glucose mg/dL	2.82 ± 11.49	−6.14 ± 22.16	5.80 ± 22.16	−14.75 ± 35.69	3.17 ± 16.99	−0.50 ± 39.83
Creatinine mg/dL	0.07 ± 0.13	0.11 ± 0.12	−0.01 ± 0.12	−0.09 ± 0.14	−0.01 ± 0.12	−0.09 ± 0.14
AST U/L	−0.60 ± 11.17	3.44 ± 10.86	3.33 ± 5.20	0.50 ± 5.47	−0.83 ± 5.78	0.88 ± 7.51
ALT U/L	6.90 ± 35.31	9.78 ± 12.53	6.67 ± 20.11	5.50 ± 23.67	2.50 ± 13.53	4.25 ± 33.88
Bilirubin mg/dL	0.24 ± 0.26	0.08 ± 0.17	−0.04 ± 0.07	−0.15 ± 0.10	0.30 ± 0.41 *	−0.07 ± 0.10 *
WBC G/L	0.37 ± 3.12	−1.57 ± 4.37	−0.37 ± 3.39	0.66 ± 1.97	−0.02 ± 3.65	−0.88 ± 4.11
LYM G/L	−0.11 ± 0.90	−0.33 ± 0.57	−0.36 ± 0.96	−0.05 ± 0.42	−0.33 ± 0.68	−0.29 ± 0.53
NEU G/L	0.51 ± 2.77	−0.95 ± 3.51	−0.01 ± 3.48	0.60 ± 2.16	0.29 ± 3.13	−0.29 ± 3.88
MON G/L	0.02 ± 0.21	−0.02 ± 0.29	−0.02 ± 0.32	−0.09 ± 0.19	0.03 ± 0.35	−0.13 ± 0.15
EOS G/L	0.02 ± 0.20	0.01 ± 0.13	0.02 ± 0.18	−0.01 ± 0.08	0.06 ± 0.24	0.00 ± 0.09
IG G/L	−0.02 ± 0.10	−0.09 ± 0.20	0.05 ± 0.20	0.06 ± 0.22	0.02 ± 0.21	−0.07 ± 0.33
BASO G/L	−0.02 ± 0.04	−0.02 ± 0.04	−0.02 ± 0.03	−0.01 ± 0.03	−0.02 ± 0.03	−0.01 ± 0.03
RBC T/L	0.02 ± 0.42	−0.09 ± 0.45	−0.14 ± 0.36	−0.23 ± 0.35	0.00 ± 0.39	−0.32 ± 0.64
HBG mmol/L	0.27 ± 0.70	−0.07 ± 0.73	−0.23 ± 0.66	−0.21 ± 0.81	0.26 ± 0.62	−0.24 ± 1.06
HCT L/L	0.01 ± 0.04	0.00 ± 0.04	−0.01 ± 0.03	−0.01 ± 0.03	0.01 ± 0.03	−0.01 ± 0.04
MCV fL	1.75 ± 2.27	1.04 ± 1.74	0.70 ± 2.69	3.85 ± 5.70	0.70 ± 2.69	3.85 ± 5.70
MCH fmol	0.05 ± 0.07	0.02 ± 0.05	0.01 ± 0.06	0.06 ± 0.09	0.01 ± 0.06	0.06 ± 0.09
MCHC mmol/L	0.22 ± 0.51	0.02 ± 0.32	−0.06 ± 0.34	−0.18 ± 0.77	0.13 ± 0.43	−0.11 ± 0.61
PLT G/L	−77.73 ± 100.23	−37.93 ± 101.16	22.00 ± 52.23	−28.30 ± 70.23	−49.08 ± 72.23	−54.50 ± 86.43
BDNF pg/mL	9.05 ± 237.11	−189.70 ± 409.98	87.73 ± 202.68	−106.78 ± 194.25	96.78 ± 407.53	−285.83 ± 455.51

* *p* < 0.05. Abbreviations: EG, exercise group; CG, control group; AST, aspartate aminotransferase; ALT, alanine aminotransferase; WBC, white blood count; LYM, lymphocytes; NEU, neutrophils; MON, monocytes; EOS, eosinophils; IG, immunoglobulins; BASO, basophils; RBC; red blood cell; HBG; hemoglobin; HCT, hematocrit; MCV, mean corpuscular volume; MCH, mean corpuscular hemoglobin; MCHC, mean corpuscular hemoglobin concentration; PLT, platelet count; BDNF, brain-derived neurotrophic factor.
